# Interleukin-4 Receptor Targeting Peptide Decorated Extracellular Vesicles as a Platform for In Vivo Drug Delivery to Thyroid Cancer

**DOI:** 10.3390/biomedicines10081978

**Published:** 2022-08-15

**Authors:** Prakash Gangadaran, Gowri Rangaswamy Gunassekaran, Ramya Lakshmi Rajendran, Ji Min Oh, Sri Murugan Poongkavithai Vadevoo, Ho Won Lee, Chae Moon Hong, Byungheon Lee, Jaetae Lee, Byeong-Cheol Ahn

**Affiliations:** 1 BK21 FOUR KNU Convergence Educational Program of Biomedical Sciences for Creative Future Talents, Department of Biomedical Sciences, School of Medicine, Kyungpook National University, Daegu 41944, Korea; 2 Department of Nuclear Medicine, School of Medicine, Kyungpook National University, Daegu 41944, Korea; 3 Department of Biochemistry and Cell Biology, Kyungpook National University, Daegu 41944, Korea; 4 Department of Nuclear Medicine, Kyungpook National University Hospital, Daegu 41944, Korea

**Keywords:** interleukin-4 receptor, extracellular vesicles, IL-4R-binding peptide, anaplastic thyroid cancer, drug delivery

## Abstract

Mesenchymal stem cell (MSC)-derived extracellular vesicles (EVs) have been demonstrated to deliver therapeutic drugs in preclinical studies. However, their use is limited, as they lack the ability to specifically deliver drugs to tumor tissues in vivo. In the present study, we propose the use of a targeting peptide, IL-4R-binding peptide (IL4RPep-1), to specifically deliver intravenously (i.v.) infused EVs to thyroid tumors. In vivo, a xenograft tumor model was treated with either the control peptide (NSSSVDK) or IL4RPep-1-Flamma; mice were fluorescently imaged (FLI) using an in vivo imaging system at 0–3 h post-treatment. EVs (labeled with DiD dye) were conjugated with IL4RPep-1 through a DOPE-NHS linker and administered to mice intravenously. FLI was performed 0–24 h post-injection, and the animals were sacrificed for further experiments. The morphology and size of EVs, the presence of EV markers such as CD63 and ALIX, and the absence of the markers GM130 and Cyto-C were confirmed. In vivo, FLI indicated an accumulation of i.v. injected IL4RPep-1-Flamma at the tumor site 90 min post-injection. No accumulation of NSSSVDK-Flamma was detected. In vivo, IL4RPep-1-EVs targeted the Cal-62 tumor 2 h post-injection. NSSSVDK-EVs were not even detected in the tumor 24 h post-injection. The quantification of FLI showed a significant accumulation of MSC-EVs in the tumor 2 h, 3 h, and 24 h post-injection. Furthermore, ex vivo imaging and an IF analysis confirmed the in vivo findings. Our results demonstrate the use of the IL4RPep-1 peptide as a targeting moiety of EVs for IL-4R-expressing anaplastic thyroid tumors.

## 1. Introduction

Extracellular vesicles (EVs) are nanosized membrane vesicles released by cells into biological fluids such as blood, urine, saliva, and cell culture medium [1,2,3,4]. EVs, also known as exosomes and microvesicles, contain proteins, lipids, miRNA, mRNA, and DNA [5,6,7,8]. Exosomes range in diameter from 50 to 300 nm and are secreted into the extracellular space by multivesicular bodies formed inside the cells. Microvesicles have diameters of 50 to 450 nm and are formed by budding from the cell membrane. EVs play a crucial role in long distance cell–cell communication without requiring direct contact between cells [1,9,10,11,12]. Novel biological roles of EVs continue to be described, highlighting the importance of the functional cargos transferred from one cell to another in physiological and pathological processes.

EVs are excellent endogenous nanocarriers for exogenous drug delivery systems [13,14,15]. Recently, several studies have shown ameliorated recovery from disease with the use of drug-loaded EVs, including studies performed in different cancer models [16,17,18]. The specific delivery of anticancer drugs to tumor tissues reduces side effects and significantly improves therapeutic outcomes. Based on the findings of previous studies that compared drug delivery by intratumor and intravenous injections [16,19,20,21], intratumor drug delivery was found to be more effective [16,20]. Though intratumor injections are acceptable/feasible in preclinical studies, in a clinical setting it is not feasible to reach tumors in certain locations. In addition, intratumor injection is not practicable in treating multifocal or disseminated tumors. Thus, under clinical conditions, intravenous injection is the ideal route for drug administration in treating tumors. However, most of the intravenously (i.v.) injected exosomes are absorbed by the liver [18,22,23,24,25,26]. Indeed, a novel strategy is needed for in vivo intravenous injections of EVs loaded with drugs to specifically target tumor tissues in order to reduce the off-target systemic side-effects.

The interleukin-4 (IL-4) receptor binds to both IL-4 and IL-13 and couples it to the JAK-STAT pathway [27]. The IL-4/IL-4R interaction activates downstream signaling pathways, leading to the induction of the expression of anti-apoptotic proteins (Bcl-xL and Bcl-2) and chemotherapy-induced apoptosis [28]. The binding of IL-4 to IL-4R results in the efficient internalization of IL-4R by cells [29]. IL-4Rs are upregulated in various types of tumors, including lung carcinoma [30], colorectal carcinoma [31], breast carcinoma [32], acquired immunodeficiency syndrome (AIDS)-related Kaposi’s sarcoma [33], melanoma [32], renal cell carcinoma [34], ovarian carcinoma [32], head and neck squamous cell carcinoma [35], and glioblastoma [36]. Many studies suggest IL-4R as an effective target for cancer therapy, and it is in use in clinics for treating different types of cancer [37,38,39,40,41,42]. Various types of thyroid cancers, namely, papillary, follicular, and anaplastic, express IL-4R [43]. However, to our knowledge, currently, antitumor therapies have not targeted IL4R to treat thyroid cancers. A screening of a phage-displayed peptide library identified an IL4R-targeting peptide named IL4RPep-1, a 9-mer peptide with the sequence CRKRLDRNC, which is homologous to the sequence of IL4 [44].

The present study aims at developing a platform for delivering anticancer drugs to human anaplastic thyroid tumor tissues in a mouse model using IL4R-targeting peptide engineered EVs. EVs were derived from mesenchymal stem cells (MSCs) obtained from bone marrow. The tumor-targeting capability of EVs was conferred by the engineered IL4R-targeting peptide using a membrane phospholipid-based linker, dioleylphosphatidylethanolamine (DOPE), with a biological anchor for a membrane (BAM). BAM rapidly and effortlessly anchors the targeting ligands to the cell membrane (similar to the EV membrane) without cytotoxicity and enhances the homing of cells to target tissues [45,46]. The IL4RPep-1 extracellular vesicles (IL4RPep-1-EVs) were purified after labeling and were characterized. The enhanced tumor-targeting capability of IL4RPep-1-EVs was validated both in vitro and in vivo.

## 2. Materials and Methods

### 2.1. Cell Culture

Human anaplastic thyroid cancer cells (Cal-62), a murine breast cancer cell line (4T1), and human bone-marrow-derived MSCs were purchased (American Type Culture Collection, VA, USA) and cultured in Dulbecco’s modified Eagle’s medium (DMEM) and DMEM/F12 (Gibco, MA, USA), respectively, and supplemented with 10% fetal bovine serum (FBS) and 1% antibiotic–antimycotic mixture (Invitrogen, Carlsbad, CA, USA) at 37 °C in an atmosphere of 5% CO_2_.

### 2.2. Isolation of EVs and Purification

EVs were cultured in EV-depleted FBS produced by ultracentrifugation at 120,000× *g* for 18 h at 4 °C. EVs were isolated from culture supernatants by differential centrifugation, as described previously [47]. Briefly, the cell culture medium was sequentially centrifuged to remove cells. The cell-free supernatants were filtered through a 0.22 μM syringe filter/vacuum filter and subjected to ultracentrifugation at 100,000× *g* for 60 min at 4 °C. Pellets were washed with phosphate-buffered saline (PBS), followed by ultracentrifugation at 100,000× *g* for 60 min at 4 °C (Supplementary Figure S1). All the ultracentrifugation steps were performed using an Optima™ L-100 XP ultracentrifuge with Ultra-Clear tubes (Beckman Coulter, Indianapolis, IN, USA).

### 2.3. Western Blotting

Western blotting was performed as described previously [22]. Briefly, whole-cell or EV lysates were prepared using a radioimmune precipitation assay (RIPA) buffer (Thermo Scientific, Waltham, MA, USA) and a protease inhibitor cocktail (Sigma-Aldrich, St. Louis, MO, USA). Equal amounts of protein samples were loaded and separated by 10% sodium dodecyl sulfate-polyacrylamide gel electrophoresis (SDS-PAGE). The separated proteins were electrotransferred to a polyvinylidene (PVDF) membrane (Millipore, Burlington, MA, USA) and probed with the primary antibody against the target protein, followed by a horseradish peroxidase (HRP)-conjugated secondary antibody. Antibody-labeled proteins were detected using an enhanced chemiluminescence kit (GE Healthcare, Chicago, IL, USA) according to the manufacturer’s protocol. All antibodies used in this study are listed in Table S1.

### 2.4. Peptides

IL4RPep-1 (CRKRLDRNC) peptides were synthesized by Peptron Inc. (Daejeon, Korea). NSSSVDK, a peptide sequence present in the phage coat protein, was used as a control peptide [29].

### 2.5. Labeling of EVs with Peptide Using DOPE-BAM

DOPE-BAM (M.W. 744), composed of dioleylphosphatidylethanolamine (DOPE), 80 units of methoxy polyethylene glycol (PEG), and succinyl-N-hydroxy-succinimidyl (NHS) ester, was kindly provided by Dr. Terayuki Nagamune (The University of Tokyo, Japan). Peptides were conjugated to the NHS end of the DOPE biocompatible anchor for membrane (BAM) by incubating 95 μL of 84.5 μM peptide (IL4RPep-1 (CRKRLDRNC) or control (NSSSVDK)) solution in PBS with 5 μL of 15 mM DOPE-BAM solution in dimethyl sulfoxide (DMSO) for 1 h at 37 °C. Tris-HCl buffer (1 M, pH 8.0) was used as a stop solution to terminate the reaction. The reaction mixture was subjected to dialysis to remove unconjugated peptides, and the dialysate was stored at −20 °C until further use. Peptide labeling was achieved by incubating 100 µg of DiD (a lipophilic dye, 1,1′-dioctadecyl-3,3,3′,3′-tetramethylindodicarbocyanine perchlorate, Invitrogen)-labeled EVs or unlabeled EVs with 10 μM DOPE-BAM peptide conjugate in PBS at 37 °C for 15 min. Exoquick-TC (System Bioscience, Palo Alto, CA, USA) was added to the above reaction mixture and incubated for 45 min at 4 °C, followed by centrifugation at 13,000 rpm for 3 min to remove unbound DOPE-BAM peptides. The peptide-labeled EVs are referred to as IL4RPep-1-EVs^DiD^.

### 2.6. Analysis of IL4RPep-1-EVs by Flow Cytometry

A flow cytometry analysis of EVs was performed as described previously, with modifications [48]. Briefly, EVs, IL4RPep-1-FITC-EVs (without DOPE-BAM) or IL4RPep-1-FITC-EVs (with DOPE-BAM), were attached to 4 μm aldehyde/sulfate latex beads (Invitrogen) by mixing 5 μg of EVs in 10 μL of beads for 15 min at 37 °C. PBS was added to the above mixture to make a final volume of 1 mL, followed by incubation for 2 h on a rotating wheel at 37 °C. After incubation, the reaction was terminated by the addition of 100 mM glycine and centrifuged at 15,000× *g* for 2 min. The supernatant was discarded, and the beads were resuspended in 1 mL of PBS for flow cytometry analysis. The samples were analyzed using an automated high-speed cell counter/sorter (Beckman Coulter, Indianapolis, IN, USA).

### 2.7. Transmission Electron Microscopy (TEM)

TEM was performed as described previously [47]. Briefly, EV pellets, IL4RPep-1-EVs^DiD^ and NSSSVDK-EVs^DID^, were resuspended in 2% paraformaldehyde (PFA) overnight at 4 °C. A drop (10 μL) of the suspension was placed onto a formvar-carbon-coated EM grid surface for 20 min. EVs on the grid were washed with a 100 μL droplet of PBS that was pipetted on a sheet of parafilm. The grids were inverted and placed over PBS using clean forceps. For fixation, the grids were transferred onto a 50 μL drop of 1% glutaraldehyde for 5 min. The grids were then stained with 2% uranyl acetate for 5 min, a negative-staining solution. The grids were then washed with distilled water seven times for 2 min. EVs were observed using an HT7700-TEM (Hitachi, Tokyo, Japan).

### 2.8. Nanoparticle Tracking Analysis (NTA)

An NTA was performed using a NanoSight LM10 (Malvern, UK) equipped with a 640 nm laser and a fluoroelastomer O-ring (Viton), as described previously [47]. EVs, IL4Rpep-1-Evs^DiD^ and NSSSVDK-Evs^DID^, suspended in PBS were diluted 1000-fold with Milli-Q water. The diluted samples were injected into the sample chamber and measured. Measurements were performed in triplicate, and each measurement was performed on 60 sections. Values corresponding to the measured particle size were analyzed using NanoSight software (Malvern, UK).

### 2.9. Immunofluorescence (IF) Assay

IF was performed as described previously [49]. Briefly, Cal-62 cells were seeded in 4-well chamber slides. After 24 h of culturing, cells were fixed in chilled 100% methanol at −20 °C, blocked with 5% bovine serum albumin (BSA) for 1 h, and incubated with an antimouse IL-4Rα antibody in 5% BSA for 1 h, followed by incubation with an Alexa Fluor-647-antimouse secondary antibody for 1 h. The slides were mounted with coverslips using Vectashield antifade mounting medium with 4′,6-diamidino-2-phenylindole (DAPI) (Vector Laboratories, Peterborough, UK) and sealed. Confocal images were obtained using an LSM 800 confocal laser scanning microscope (Carl Zeiss, Baden-Württemberg, Germany).

### 2.10. Cellular Binding of Peptides

The cellular binding of the IL4R-Pep-1 peptide was analyzed by incubating the IL4RPep-1-FITC and NSSSVDK-FITC peptides with Cal-62 for 1 h at 37 °C under 5% CO_2_, followed by fixation in methanol. Slides were mounted using Vectashield antifade mounting medium with DAPI (Vector Laboratories, Peterborough, UK) and sealed. The cellular binding of peptides was observed using an LSM 800 confocal laser scanning microscope (Carl Zeiss, Baden-Württemberg, Germany). Cal-62 and 4T1 cells were incubated with 10 mM FITC-labeled peptides for 1 h at 4 °C. After incubation, cells were washed twice (PBS) to remove unbound FITC-labeled peptides and analyzed by flow cytometry using an automated high-speed cell counter/sorter.

### 2.11. Cytotoxicity Assay

Cells were seeded (5 × 10^3^ cells per well) in triplicate in 96-well plates with 100 μL of complete medium together with 0, 10, 20, and 40 μM/mL of NSSSVDK or IL4R-Pep-1. Cell proliferation was measured after 24 h using the cell counting kit (CCK)-8 assay (Dojindo Laboratories, Kyushu, Japan). After 24 h, 10 μL of CCK-8 solution was added to each well, and the plates were incubated in a CO_2_ incubator for 2 h at 37 °C. Optical densities (OD) were determined using an enzyme-linked immunosorbent assay (ELISA) plate reader according to the manufacturer’s instructions.

### 2.12. In Vitro Internalization of EVs by Cal-62

To analyze the internalization of EVs in thyroid cancer cells, 20 μg of IL4RPep-1-EVs^DiD^, EVs^DiD^, or EVs was incubated with DiD (1,1′-dioctadecyl-3,3,3′,3′-tetramethylindodicarbocyanine, 4-chlorobenzenesulfonate salt, Invitrogen) for 20 min at 37 °C, washed with PBS, and subjected to a two-step OptiPrep density gradient ultracentrifugation, as described above. Cal-62 cells grown on chamber slides were incubated with DiD-labeled EVs for 1 h, 2 h, 3 h, and 4 h at 37 °C in 5% CO_2_. After incubation, the cells were fixed in methanol, mounted using an antifade mounting medium with DAPI, and sealed. The cellular internalization of EVs was observed using an LSM 800 confocal laser scanning microscope (Carl Zeiss, Baden-Württemberg, Germany).

### 2.13. In Vivo Targeting of IL4RPep-1 to Cal-62 Xenograft Tumor in Mice

All described animal experimental procedures were performed in accordance with the Guiding Principles for the Care and Use of Laboratory Animals. Cal-62 cells (5 × 10^6^) were injected subcutaneously into the right lower flank region of nude mice and allowed to grow for six weeks, and the mice were used for further experiments. The mice were anesthetized with 2.5% isoflurane, and NSSSVDK-Flamma or IL4RPep-1-Flamma (10 μM/mL) were injected intravenously (i.v.). Fluorescent images were captured using the IVIS III imaging system after 45, 90, 120, and 180 min and 24 h post-injection. Fluorescent signals were captured and quantified using the IVIS III imaging system software (Living Image Software, PerkinElmer, Waltham, MA, USA).

### 2.14. In Vivo Targeting of IL4RPep-1-EVs^DiD^ to Cal-62 Xenograft Tumor in Mice

Mice (n = 3) were anesthetized with 2.5% isoflurane, and IL4RPep-1-EVs^DiD^ or NSSSVDK-EVs^DID^ (50 μg) were injected i.v. Fluorescent images were captured using the IVIS III imaging system after 0, 1, 2, 3, and 24 h. Tumors were excised, and ex vivo fluorescent images were captured and quantified using the IVIS III imaging system software.

### 2.15. Immunofluorescent Analysis (IFA) of Tissues

Fresh tumor tissues were fixed in 4% PFA at 4 °C. The tissue specimens were transferred to 15% sucrose (Sigma) in PBS for 6–12 h (until tissues sank) and then transferred to 30% sucrose in PBS overnight. The tissues were embedded in OCT compound (Sigma-Aldrich, St. Louis, MO, USA) and cryo-sectioned, and IFA was performed as described previously [50].

### 2.16. EV-TRACK Knowledgebase

All relevant experimental data were submitted to the EV-TRACK knowledgebase (EV-TRACK ID: EV180049) [51].

### 2.17. Statistical Analysis

Data are expressed as means ± standard deviations (SD). The differences between groups were determined by Student’s *t*-test. Statistical analyses were performed using GraphPad Prism 7 (version 7.04; GraphPad Software Inc., San Diego, CA, USA). Significance was defined as: *p* < 0.05 (*), *p* < 0.01 (**), and *p* < 0.001 (***).

## 3. Results

### 3.1. Characterization of EVs

**Figure 1:**
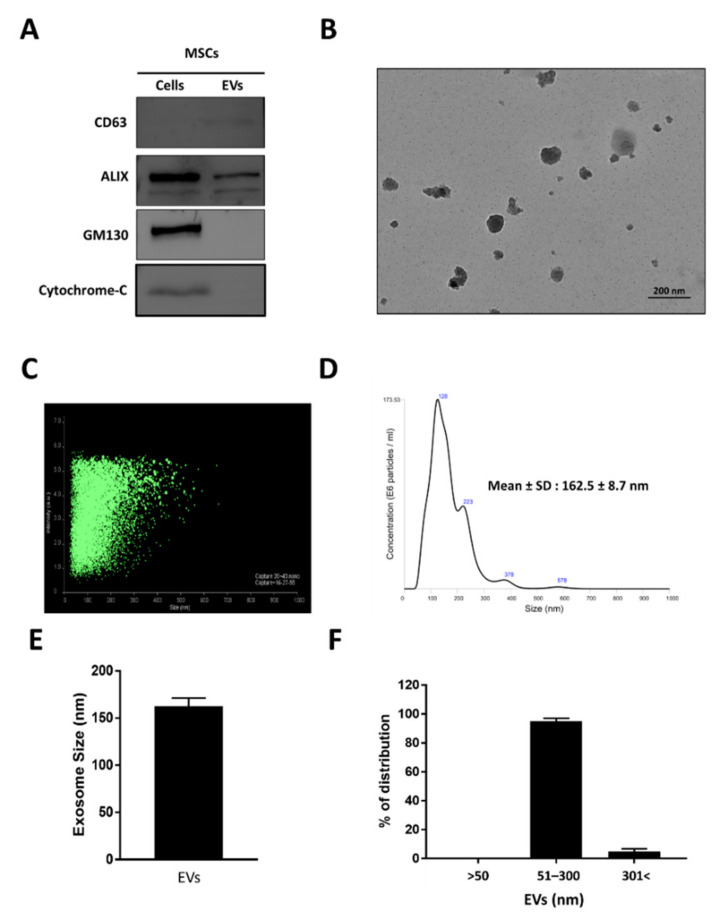
Isolation and characterization of extracellular vesicles (EVs) derived from mesenchymal stem cells (MSCs). (**A**) Western blot analysis of cell compartment markers in MSCs and EVs. Antibodies specific for CD63, ALIX, GM130, and cytochrome-C were used for the study. (**B**) Transmission electron microscopy (TEM) analysis confirming the morphology of EVs (scale bar: 200 nm). (**C**) Scatter plot of EVs analyzed by nanoparticle tracking analysis (NTA). (**D**,**E**) Size of EVs determined by NTA (average diameter: 162.5 ± 8.7 nm). (**F**) Percentage distribution of EVs is represented on a bar graph (approximately 95% of EVs ranged in size between 50 and 300 nm). Data are represented as means ± standard deviations (SD).

EVs were isolated from human MSCs using an ultracentrifugation method, as described previously with modifications [47] (Supplementary Figure S1). Isolated EVs were subjected to a Western blot analysis to confirm the expression of EVs and cell markers present in EVs and MSCs lysates, respectively. The Western blot results showed enriched protein bands for both CD63 (a membrane-associated tetraspanin family protein) and Alix (a cytoplasmic ubiquitin protein) in EVs compared to MSCs, which were positive for Alix but lacked CD63. Furthermore, EVs were found to be negative for the cell markers GM130 (Golgi-apparatus-associated protein) and cytochrome-C (mitochondrial protein) compared to MSCs, which were positive for both these cell markers. The presence of glyceraldehyde 3-phosphate dehydrogenase (GAPDH) was detected in both EVs and MSCs (Figure 1A). Typical morphological features of EVs were confirmed in the isolated EV fraction using TEM. The TEM analysis revealed the presence of characteristic spherical vesicles of approximately 150 nm in diameter specific to EVs (Figure 1B). The size and distribution of EVs isolated from MSCs were further probed using NTA. An NTA scattering plot showed the presence of clustered EVs ranging in size from 30 to 300 nm (Figure 1C). The highest peak was visualized at 123 nm, indicating the size of the EVs to be 162.5 ± 8.7 nm, ranging from 50 to 350 nm (Figure 1D,E). Furthermore, most of the EVs ranged in size between 50 and 300 nm, which included ~95% of the total EV fraction (Figure 1F). Taken together, these results confirm the successful isolation and enrichment of EVs from cell culture supernatants without cell or cell organelle contamination.

### 3.2. Labeling of EVs with IL4RPep-1

**Figure 2:**
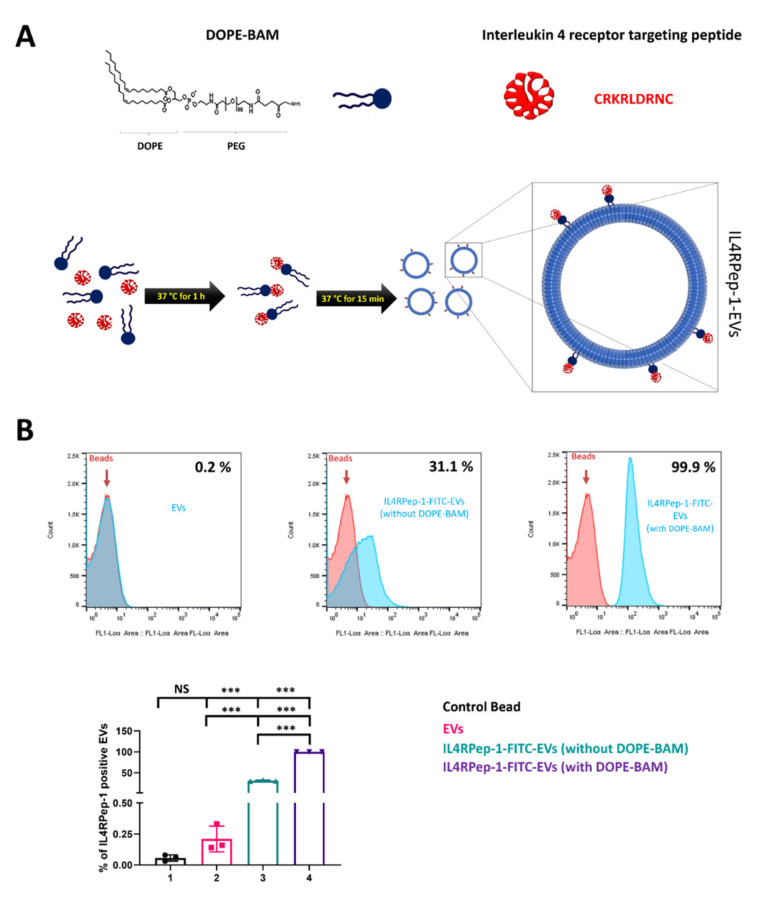
Labeling of extracellular vesicles with IL4RPep-1. (**A**) DOPE biological anchor for membrane (BAM), composed of dioleylphosphatidylethanolamine, methoxy polyethylene glycol (PEG), and succinyl-N-hydroxy-succinimidyl (NHS) ester conjugated with peptide and peptide complex, was inserted into the membranes of EVs. (**B**) Flow cytometry analysis of EVs and EVs labeled with IL4RPep-1-FITC in the presence and absence of DOPE-BAM complex. Percentage of EVs labeled with IL4RPep-1 is represented on a bar graph. Data are represented as means ± standard deviations (SD). Statistical differences between groups were analyzed using Student’s *t*-test. *** *p* < 0.001.

To generate IL-4R-targeted EVs, EVs isolated from human MSCs were labeled with IL4RPep-1 using a DOPE-BAM linker. IL4RPep-1-FITC was conjugated through its primary amine to DOPE-BAM to form IL4Rpep-1-labeled EVs in the form of DOPE-BAM-IL4RPep-1-FITC, represented in the present study as IL4Rpep-1-EVs (Figure 2A). Correspondingly, the control peptides labeled with EVs are represented as NSSSVDK-EVs. The labeling of peptides to EVs was confirmed by a flow cytometry analysis. Our results indicated the percentage of IL4RPep-1-FITC with DOPE-BAM bound to EVs to be approximately 99.9% compared to IL4RPep-1-FITC without DOPE-BAM bound to EVs, which was approximately 31.1% (Figure 2B). DOPE-BAM significantly enhanced the binding efficiency of IL4RPep-1-FITC to EVs (*p* < 0.001), as DOPE-BAM acts as a linker between EVs and IL4RPep-1. Control beads and beads with bound EVs without labeling were negligible (<0.2%; Figure 2B). Taken together, these results indicate the successful labeling of EVs with IL4RPep-1 through a DOPE-BAM linker.

### 3.3. Morphology, Size, and Distribution of EVs after IL4RPep-1 Labeling

**Figure 3:**
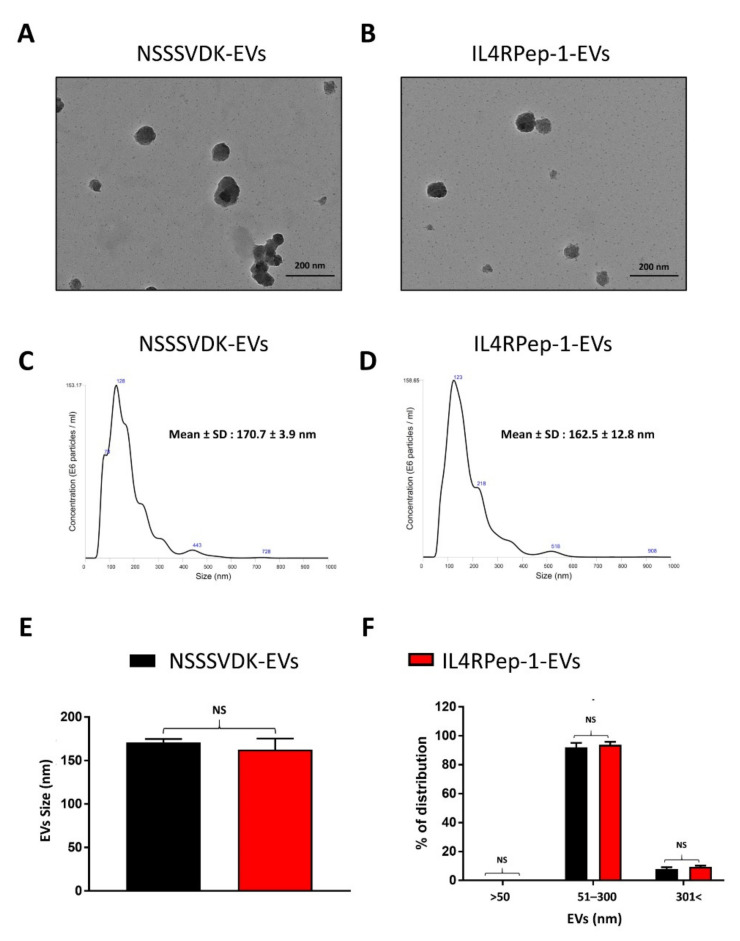
Peptide labeling maintains the morphology, size, and distribution of extracellular vesicles (EVs). (**A**,**B**) Morphology of NSSSVDK-EVs and IL4RPep-1-EVs confirmed by transmission electron microscopy (TEM; scale bar: 200 nm). (**C**–**E**) Size of NSSSVDK-EVs and IL4RPep-1-EVs determined by nanoparticle tracking analysis (NTA) (Diameters: NSSSVDK-EVs, 170.7 ± 3.9 nm; IL4RPep-1-EVs, 162.5 ± 12.8 nm). (**F**) Percentage distribution of NSSSVDK-EVs and IL4RPep-1-EVs represented on bar graph (approximately 95% of EVs ranged in size between 50 and 300 nm). Data are represented as means ± standard deviations. Statistical differences between groups were analyzed using Student’s *t*-test.

To study the effect of peptide labeling on the structure of EVs, a TEM analysis was performed on EVs, IL4RPep-1-EVs, and NSSSVDK-EVs. The TEM results revealed no significant differences in classical morphology (round/spherical) between IL4RPep-1-EVs and NSSSVDK-EVs (Figure 3A,B). Comparably, the results of the NTA analysis indicated no significant variation in the size of EVs among naïve EVs, IL4RPep-1-EVs, and NSSSVDK-EVs (Figure 3C–E, Supplementary Figure S2A,B). Consistently, the results of the size distribution analysis of EVs revealed no significant difference in size distribution between IL4RPep-1-EVs and NSSSVDK-EVs (Figure 3F, Supplementary Figure S2C). Taken together, our results confirm that the peptide labeling of EVs retains the morphology, size, and distribution of EVs.

### 3.4. In Vitro, Expression of IL4Rα in Thyroid Cancer Cells and Preferential Binding of IL4RPep-1 to IL4Rα-Expressing Cancer Cells

**Figure 4:**
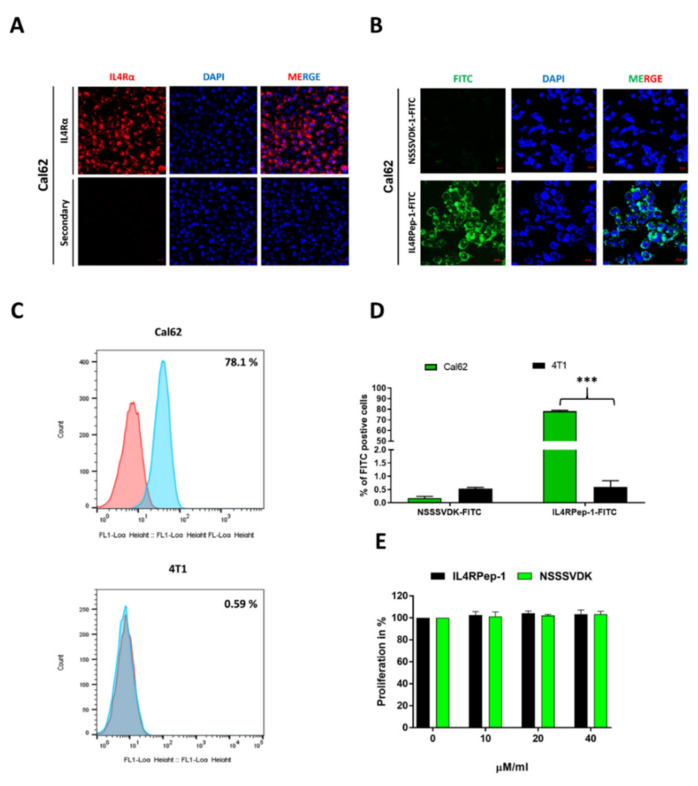
Expression of interleukin-4 receptor (IL4R) in thyroid cancer cells and preferential binding of IL4RPep-1 to IL4Rα-expressing cancer cells in vitro. (**A**) Immunofluorescent imaging of Cal-62 using specific IL4Rα antibody (scale bar: 20 µm). (**B**) Immunofluorescent imaging of Cal-62 cells incubated with NSSSVDK-FITC and IL4RPep-1-FITC (scale bar: 20 µm). (**C**) Flow cytometry analysis of Cal-62 and 4T1 cells incubated with NSSSVDK-FITC (red) and IL4RPep-1-FITC (blue). (**D**) Percentage of peptide-bound cells is represented on a bar graph. (**E**) Cell viability of Cal-62 cells incubated with 0, 10, 20, and 40 µM/mL of NSSSVDK and IL4RPep-1 24 h post-incubation. Data are represented as means ± standard deviations (SD). Statistical differences between groups were analyzed using Student’s *t*-test. *** *p* < 0.001.

The expression of IL4Rα was analyzed in the human thyroid cancer cell line Cal-62. The results of the immunofluorescence assay indicated the aberrant expression of IL4Rα in the cytoplasm and membrane of Cal-62 cells (Figure 4A). Targeted drug delivery based on IL4R is dependent on the ability of IL4RPep-1 to bind to IL4R-expressing cells. The incubation of Cal-62 cancer cells with IL4RPep-1-FITC and NSSSVDK-FITC resulted in the rapid binding of IL4RPep-1-FITC to the cell surface of Cal-62 cells (Figure 4B). A flow cytometry analysis was performed for immunofluorescent imaging. The results of the flow cytometry confirmed significant binding of IL4RPep-1-FITC (~78.17%) to Cal-62 cells compared to that of NSSSVDK-FITC (~0.17%), where no binding was detected. Both IL4RPep-1-FITC (~0.59%) and NSSSVDK-FITC (~0.53%) showed no binding in 4T1 cells (In vitro IL4R-expression-lacking mouse breast cancer cells [52]) (Figure 4C). Accordingly, IL4RPep-1-FITC could significantly bind to Cal-62 (*p* < 0.001) but not to 4T1 cells (Figure 4D). Furthermore, the effect of IL4RPep-1 or NSSSVDK binding on the viability/proliferation of Cal-62 cells was tested. Our results revealed no significant effect of IL4RPep-1 or NSSSVDK (10–40 µM/mL) binding on the viability/proliferation of cells (Figure 4E). The concentration of the respective peptides used in all our experiments was 10 µM/mL.

### 3.5. Enhanced Internalization of IL4RPep-1-EVs into Cal-62 Cells by IL4RPep-1 Labeling

**Figure 5:**
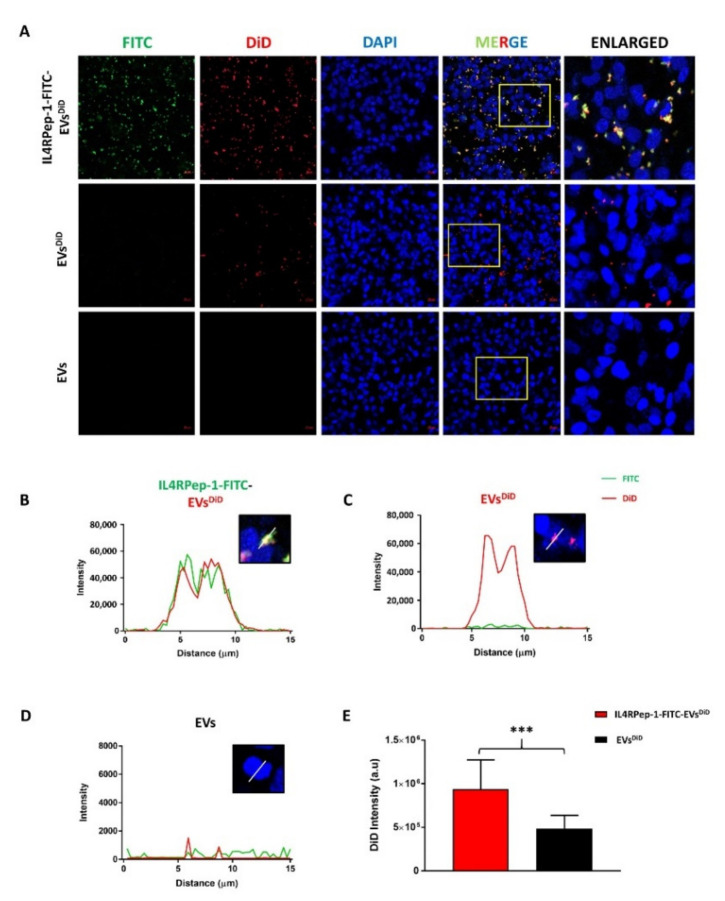
Internalization of IL4RPep-1-EVs by Cal-62 cells. Enhanced internalization of extracellular vesicles (EVs) by IL4RPep-1. (**A**) Immunofluorescent imaging of Cal-62 cells, 1 h post-incubation with 20 μg of IL4RPep-1-FITC-EV ^DiD^, EV ^DiD^, and EVs; IL4RPep-1 (green) and EVs (red) (scale bar: 20 µm). (**B**–**D**) Fluorescent intensity analysis of IL4RPep-1-FITC-EV ^DiD^, EV ^DiD^, and EVs for FITC and DiD by Zen 2.3 software (Zeiss, Baden-Württemberg, Germany) from (**A**) and represented on the graph in arbitrary units. (**E**) Fluorescent intensity analysis of IL4RPep-1-FITC-EV ^DiD^ and EV ^DiD^ for DiD signals from (**A**) by ImageJ 1.52a software (Wayne Rasband, Maryland, USA), represented on a bar graph. Data are represented as means ± standard deviations (SD). Statistical difference between groups was analyzed using Student’s *t*-test. *** *p* < 0.001.

Successful drug delivery requires the internalization of EVs. Accordingly, the internalization of IL4RPep-1-labeled EVs and unlabeled EVs into Cal-62 cells was analyzed by fluorescent imaging. EVs labeled with DiD (a lipophilic fluorescent dye) were linked to IL4RPep-1-FITC (represented as IL4RPep-1-FITC-EVs^DiD^) and incubated with Cal-62. Fluorescent microscopic imaging revealed the internalizations of both IL4RPep-1-FITC-EVs^DiD^ and EVs^DiD^ into Cal-62 cells (Figure 5A). In addition, co-localization of DiD (EVs) and FITC signals (IL4RPep) was observed (Figure 5A). In Cal-62 cells incubated with IL4RPep-1-FITC-EVs^DiD^, the intensities of the DiD and FITC signals were found to correlate with each other (Figure 5B) compared to that of Cal-62 cells incubated with EVs^DiD^, which showed only DiD but not FITC signal intensity (Figure 5C). In Cal-62 cells incubated with EVs, DiD or FITC signal intensity was either absent or detected only as a background (Figure 5D). Furthermore, microscopic imagining revealed a significantly enhanced internalization of EVs labeled with IL4RPep-1 (two-fold higher; *p* < 0.001) compared to that of unlabeled-EVs (EVs^DID^) (Figure 5E). Taken together, these results confirmed that the labeling of EVs with IL4RPep-1 could enhance the internalization of EVs by Cal-62 cells expressing IL4R.

### 3.6. In Vivo Monitoring of i.v. Injected IL4RPep-1 Targeting of Cal-62 Tumor in Mice Using Fluorescent Imaging

In vivo, the ability of IL4RPep-1 to target a Cal-62 tumor was tested prior to performing similar in vivo experiments with IL4RPep-1-labeled EVs. A xenograft tumor model was established with Cal-62 cells. Tumor-bearing mice were i.v. injected with IL4RPep-1-Flamma or NSSSVDK-Flamma peptides and imaged using the IVIS Lumina III imaging system at 45, 90, 120, and 180 min and 24 h (Supplementary Figure S3A,B). Fluorescent imaging at 45 min following i.v. injection of fluorophore-labeled peptides indicated a whole-body distribution of the peptides in mice (Supplementary Figure S3C). Imaging at 90 min post-injection revealed the localized presence of IL4RPep-1-Flamma in the Cal-62 tumor (Supplementary Figure S3C), but NSSSVDK-Flamma signals were not detected. Imaging at 120 min post-injection revealed the highest accumulation of IL4RPep-1-Flamma in the tumor, followed by 180 min and NSSSVDK-Flamma, which showed no signal in the mouse (Supplementary Figure S3C). Imaging at 24 h post-injection revealed a small accumulation of IL4RPep-1-Flamma in the tumor, and no signal was observed (Supplementary Figure S3C,D). Taken together, these experimental results confirm the ability of IL4RPep-1 to target Cal-62 tumors in mice in vivo.

### 3.7. In Vivo Monitoring of i.v. Injected IL4RPep-1-EV Targeting of Cal-62 Tumor in Mice Using Fluorescent Imaging

**Figure 6:**
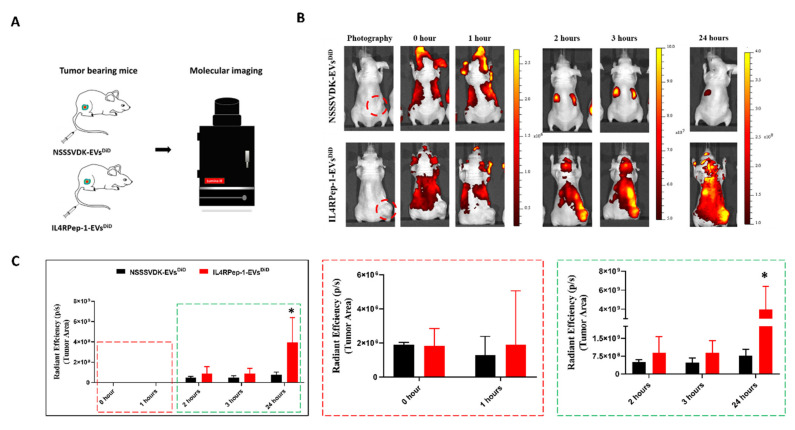
Intravenously (i.v.) injected IL4RPep-1 extracellular vesicles (EVs) target Cal-62 tumors in mice, as shown by fluorescent imaging. (**A**) Schematic image of Cal-62 tumor-bearing mouse after i.v. injection of NSSSVDK-EV^DiD^ or IL4RPep-1-EV^DiD^ (50 μg of EVs) using IVIS imaging system. (**B**) Florescent imaging at 0 h, 1 h, 2 h, 3 h, and 24 h after i.v. injection of NSSSVDK-EV^DiD^ or IL4RPep-1-EV^DiD^ in Cal-62 tumor-bearing mice (n = 3). (**C**) Quantification of florescent signals at the tumor region. Data are represented as means ± standard deviations (SD). Statistical differences between groups were analyzed using Student’s *t*-test. * *p* < 0.05.

The established Cal-62/tumor-bearing mice were divided into two groups, namely, IL4RPep-1-EVs^DiD^ and NSSSVDK-EVs^DiD^. EVs^DiD^ labeled with IL4RPep-1 or NSSSVDK were injected i.v. through the tail vein of mice bearing Cal-62 tumor and were fluorescence imaged using the IVIS Lumina III imaging system (Figure 6A). At 0 h (immediately following injection) and 1 h post-injection, fluorescence imaging indicated the rapid distribution of IL4RPep-1-EVs^DiD^ and NSSSVDK-EVs^DiD^ in the animal body. Fluorescent imaging 2 h post-injection showed targeted and enhanced accumulation (2-fold) of IL4RPep-1-EVs^DiD^ at the tumor site compared to NSSSVDK-EVs^DiD^, which was predominantly localized in the liver and spleen (Figure 6B,C). Fluorescent imaging 3 h post-injection revealed enhanced localization (2-fold) of IL4RPep-1-EVs^DiD^ signals at the tumor site compared to NSSSVDK-EVs^DiD^, which showed reduced signal intensity, with no signals detected in the tumor (Figure 6C). Fluorescent imaging 24 h post-injection indicated an overall decrease in NSSSVDK-EVs^DiD^ in mice (Figure 6B). Significantly enhanced signals were detected in the tumor following IL4RPep-1-EVs^DiD^ injection compared to that of NSSSVDK-EVs^DiD^ (*p* < 0.05; Figure 6B,C). These results suggest that the labeling of EVs with IL4RPep-1 could significantly enhance the ability of EVs to target tumors in a mouse model bearing Cal-62 tumors.

### 3.8. Ex Vivo Fluorescent and Microscopic Imaging of IL4RPep-1-EVs Using Cal-62 Tumor

**Figure 7:**
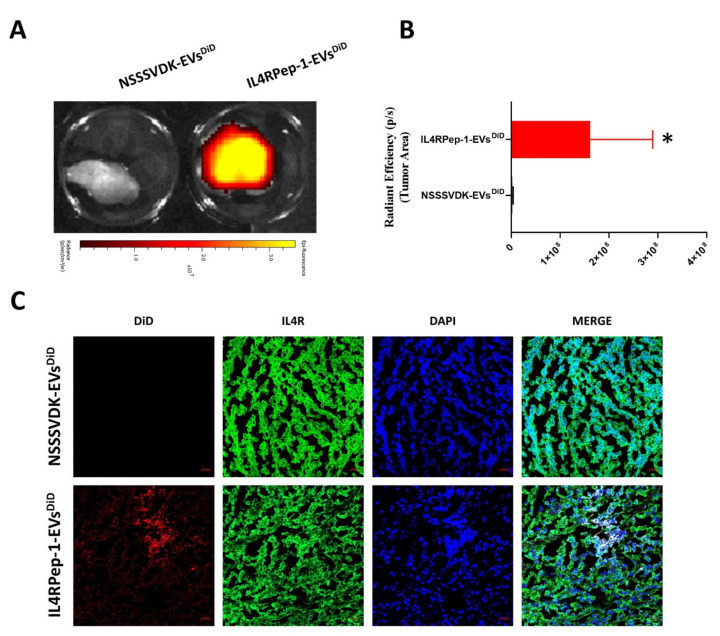
Ex vivo fluorescent and microscopic imaging of IL4R Pep-1 extracellular vesicles (EVs) in Cal-62 tumors in mice. (**A**) Ex vivo fluorescent imaging of intravenously (i.v.) injected NSSSVDK-EV^DiD^ and IL4RPep-1-EV^DiD^ in Cal-62 tumor-bearing mice. (**B**) Quantification of florescent signals present in the ex vivo tumors (n = 3) represented in (**A**). (**C**) Immunofluorescent imaging of DiD (red) and IL4R (green) in sectioned tumor (scale bar: 20 µm). Data are represented as means ± standard deviations. Statistical difference between groups was analyzed using Student’s *t*-test. * *p* < 0.05.

Tumor tissues were collected 24 h post-EV-injection and analyzed to confirm the in vivo findings and the ability of IL4RPep-1-EVs^DiD^ to target the tumor. Cal-62 tumors excised from IL4RPep-1-EVs^DiD^- and NSSSVDK-EVs^DiD^-injected mice were fluorescently imaged using the IVIS Lumina III imaging system. DiD signals were observed in ex vivo tumors obtained from mice injected with IL4RPep-1-EVs^DiD^ compared to those of NSSSVDK-EVs^DiD^-injected mice, where no signals were detected (Figure 7A). Data representing the quantification of signals revealed an enhanced accumulation of IL4RPep-1-EVs^DiD^ (*p* < 0.05) in the tumor compared to NSSSVDK-EVs^DiD^ (Figure 7B). Furthermore, a histologic analysis of the tumor tissues confirmed that IL4RPep-1-EVs^DiD^ preferentially targeted IL4R-expressing cancer cells. The signals of IL4RPep-1-EVs^DiD^ and IL4R were co-localized at the tumor tissues, as revealed by the staining of tumor sections (Figure 7C). Taken together, these results indicate that IL4RPep-1 could selectively target IL4R-expressing tumor (Cal-62) cells.

## 4. Discussion

The aim of therapeutic research is to improve patient outcomes. Accordingly, the therapeutic efficacy of the drug needs to be enhanced by increasing the drug concentration in the target tissue. Thus, targeted drug delivery systems that mitigate the toxicity and side-effects of drugs by protecting healthy tissues are currently needed [53,54]. In the field of oncology, the magnitude of adverse drug reactions may be mild and self-limited or severe, permanent, and potentially life-threatening [53]. Nanocarriers desirably shift the tissue distribution of the entrapped chemotherapeutic drug and have been employed as an alternative drug delivery system in oncology to reduce the adverse drug reactions associated with anticancer drugs to improve the efficacy and safety of the drug [18,21,55]. Recently, several studies have reported the use of EVs as nanocarriers for anticancer therapies [31,56,57,58]. As EVs can be obtained from human cells, they seem to be more appropriate drug delivery vehicles. EVs have several advantages as nanocarriers, including their ability to escape degradation and clearance by the immune system, tissue penetration facilitated by their nanosize, and prolonged circulation time in the blood because of their minimal negative zeta potential [59,60,61]. In the present study, we isolated EVs from human MSCs for targeted drug delivery to the tumor tissue. An IL4R-targeting peptide (IL4RPep-1) was labeled onto the surface of EVs, and the ability of IL4Rpep-1-labeled EVs to target the tumor was characterized and evaluated both in vitro and in vivo.

EVs were isolated from human MSCs by ultracentrifugation. Isolated EVs were positive for EV markers (CD63 and ALIX) and negative for cell markers (GM130 and cytochrome C), confirming their purity and lack of cellular contamination, comparable to other studies. TEM images revealed the morphology of isolated EVs as being intact, an important aspect that is required for preserving the biophysical and functional properties necessary for their use in drug delivery as nanocarriers [47,62]. The NTA results were comparable to previous studies [62], and accordingly, EVs ranged in size between 50 and 450 nm, with an average size of 162 nm. Collectively, these results confirmed the successful isolation of intact EVs from MSCs.

Furthermore, we exploited the use of EVs that were non-genetically modified with an IL-4R-targeting peptide, IL4RPep-1, using DOPE-BAM to target IL-4Rs in anaplastic thyroid cancer. IL4RPep-1 was found to bind to EVs in the absence of DOPE-BAM. However, in the presence of DOPE-BAM, 99.9% of EVs were labeled with IL4RPep-1. For the labeling of IL4RPep-1 to isolated EVs, EVs labeled with NSSSVDK peptide were used as a control. The morphology, intactness, size, and distribution of the labeled EVs were characterized. The labeling method did not alter the morphology of EVs, as labeled and unlabeled EVs demonstrated spherical morphology and were intact. Previously, a similar study wherein a cardiac homing peptide was labeled onto an exosome [63] was reported to have failed, as EVs need to be intact to preserve their therapeutic ability and drug-entrapping function. Furthermore, the size and distribution of EVs were not altered by the labeling procedure and were in accordance with the previous studies [31,63].

Only few studies have been reported that have used non-genetic peptide labeling of EVs (or exosomes) to target specific tissues/tumors [64,65]. One study had labeled a cardiac homing peptide to MSC exosomes to target myocardial infarction in a rat model [63]. In another study, immature mouse dendritic cells were used to produce exosomes that, after the genetic modification of a membrane protein (Lamp2b), could bind to an αv-integrin-specific iRGD peptide (CRGDKGPDC) [31]. However, this approach was feasible for use in preclinical studies but had limited application in clinical translation, as the genetic modification could alter cellular nature and could rarely transform normal cells into cancerous cells, and EVs are well-known to reflect the origin of their derived cells [22,47]. EVs can themselves be used as therapeutic agents for cancer therapy, as they carry anticancer proteins or miRNA from the cells of their origin [66,67]. The approach used in the current study is safe and feasible for clinical translation in the near future, as it does not involve genetic modification.

Most types of the cancerous cells (lung, colorectal, breast, melanoma, renal cell, ovarian, head/neck cancers, and glioblastoma) express IL4R. A number of studies have reported the expression of IL4R in all types of thyroid cancers [43,68]. However, only a single study has reportedly used IL-4R for the targeted delivery of IL-4-Pseudomonas exotoxin in mice [68]. In the present study, we chose anaplastic thyroid cancer as the target tumor because of the associated aggressiveness and poor survival [69]. In vitro, IL4R is expressed at high levels on the cell surface of Cal-62 anaplastic thyroid cancer cells. In the present study, IL4RPep-1 showed efficient binding to Cal-62 cells, and the control peptide, NSSSVDK, did not bind. The results were further confirmed by a flow cytometry analysis. The binding of IL4RPep-1 to Cal-62 cells did not affect cellular viability. These results suggest the ability of IL4RPep-1 to rapidly bind to Cal-62 cells.

In the present study, we demonstrated the internalization of IL4RPep-1-EVs and NSSSVDK-EVs by Cal-62. IL4RPep-1-EVs were rapidly internalized by Cal-62 cells compared to NSSSVDK-EVs, which were internalized at a relatively low level, up to 60 min post-incubation, confirming the ability of IL4RPep-1-EVs to target IL4R-expressing Cal-62 cancer cells. Previous studies [31,70] and the results of our study suggest IL4RPep-1-EVs as promising candidates for specifically transporting therapeutic drugs to IL4R-expressing tumors. Furthermore, this study demonstrates the stability of the binding of EVs to IL4RPep-1. In vitro, after the internalization of EVs labeled with DiD and IL4RPep-1 labeled with FITC, DiD (red) and FICT (green) were found to co-localize in Cal-62 cells. Our data demonstrate the stable binding of EVs, an aspect that was not successfully addressed in a previous study [63].

Though the in vitro data demonstrated the ability of IL4RPep-1 labeling to enhance EV affinity and internalization in IL4R-expressing Cal-62 cells, demonstrating the in vivo ability of the labeling to target tumors is of greater importance to elucidate the therapeutic effects of IL4RPep-1-labeled EVs. The biodistribution was monitored at various time points using FLI. Our data demonstrated an accumulation of IL4RPep-1-Flamma in the tumor area within 30 min upon i.v. injection compared to NSSSVDK-Flamma, which did not show accumulation at the tumor site. These results suggest both in vitro and in vivo affinity/homing of IL4RPep-1 to Cal-62 cells, corresponding to the results of previous studies [52,71]. Here, we present the possibility of using IL4RPep-1-EVs as a therapeutic modality or therapeutic drug carrier for targeting aggressive human anaplastic thyroid cancer. Our results indicated that IL4RPep-1-EVs could efficiently target tumors in a Cal-62 xenograft mouse model compared to NSSSVDK-EVs, which failed to exhibit targeted tumor specificity. Furthermore, the 24 h imaging results demonstrated the highest intensity of the signal in the tumor tissue, indicating the efficiency of IL4RPep-1-EVs in targeting the tumor. Signals were not detected in the tumors of NSSSVDK-EV-injected mice. These data suggest the use of the tumor-targeting ability of IL4RPep-1-EVs as a superior drug delivery approach in IL4R-expressing tumors, including anaplastic thyroid cancer. A similar approach with a cardiac homing peptide was used for delivering EVs to infracted hearts. The study could demonstrate only the ex vivo homing of the cardiac homing-peptide-labeled EVs to infracted hearts but failed to demonstrate in vivo homing capacity [63]. In the present study, our ex vivo data were further supported by in vivo experimental findings. As expected, a florescent microscopic imaging analysis demonstrated the efficient internalization of IL4RPep-1-EVs in IL4R-expressing tumors. Collectively, these findings indicate the feasibility of using IL4RPep-1-EVs as nanocarriers for delivering therapeutic agents in treating anaplastic thyroid cancer and other cancers that express IL4R. In addition, IL4R is also expressed on atherosclerosis, and it can be used for the treatment of vascular disease [44].

Over the past decade, increased efforts have been put into designing and developing effective EVs for treating various diseases [31,47,58]. A recent study demonstrated the enhanced therapeutic effects of MSC-derived exosomes related to radiotherapy in tumors and the metastatic tumor foci [72]. Exosomes derived from dendritic cells could directly kill tumor cells by triggering the activation of caspase and apoptosis [73]. Recently, we demonstrated that natural killer cell derived exosomes could inhibit the growth of various tumors, both in vitro and in vivo [66]. The anticancer effects of various therapeutic EVs could be significantly enhanced by applying the approach presented in the current study, which suggests the labeling of EVs with IL4RPep-1 to enhance the ability of EVs to target tumor tissue.

## 5. Conclusions

In the present study, we developed engineered EVs with an IL4R-targeting peptide, which is a new strategy that could be applied in vivo for the treatment of cancers expressing IL4R (including anaplastic thyroid cancer). Our results indicate the labeling of EVs with IL4RPep-1 is a promising strategy for the therapeutic application of EVs and/or drug delivery EVs that target IL4R-expressing cancers.

## Data Availability

Data will be made available upon reasonable request to the corresponding author.

## Supplementary Materials

The following supporting information can be downloaded at: www.mdpi.com/article/10.3390/biomedicines10081978/s1, Figure S1: Schematic representation of isolation of extracellular vesicles (EVs) from mesenchymal stem cells (MSCs); Figure S2: Peptide labeling retains the size and distribution of extracellular vesicles (EVs); Figure S3: Intravenously (i.v.) injected IL4R Pep-1 targets Cal-62 tumor in mouse (n=1/group), monitored by fluorescent imaging; Table S1: List of antibodies.
