# Supplementary material for: Interleukin-4 Receptor Targeting Peptide Decorated Extracellular Vesicles as a Platform for In Vivo Drug Delivery to Thyroid Cancer
Source: Biomedicines. 2022 Aug 15;10(8):1978. doi: 10.3390/biomedicines10081978 (PMC9406005; doi:10.3390/biomedicines10081978)
Supplement: Supplementary file 1 [file biomedicines-10-01978-s001.zip › biomedicines-1829802-supplementary.pdf]

biomedicines

Supplementary Information

Article

Interleukin-4 Receptor Targeting Peptide Decorated Extracel-lular Vesicles as A Platform For In Vivo Drug Delivery to Thyroid Cancer

Prakash Gangadaran[1,2], Gowri Rangaswamy Gunassekaran[3], Ramya Lakshmi Rajendran[2], Ji Min Oh[2], Sri Murugan Poongkavithai Vadevoo[3], Ho Won Lee[2], Chae Moon Hong[2,4], Byungheon Lee[1,3], Jaetae Lee[2,4] and Byeong-Cheol Ahn[1,2,4,*]

1 BK21 FOUR KNU Convergence Educational Program of Biomedical Sciences for Creative Future Talents, Department of Biomedical Sciences, School of Medicine, Kyungpook National University, Daegu, Republic of Korea.
2 Department of Nuclear Medicine, School of Medicine, Kyungpook National University, Daegu, Republic of Korea.
3 Department of Biochemistry and Cell Biology, Kyungpook National University, Daegu, Republic of Korea.
4 Department of Nuclear Medicine, Kyungpook National University Hospital, Daegu, Republic of Korea.
* Correspondence: Byeong-Cheol Ahn (abc2000@knu.ac.kr); Tel: 82-53-420-5583.

Table-S1. List of antibodies.

S. no	Antibody	Company	WB	IF
1	CD63	Abcam	1:4000	-
2	ALIX	Abcam	1:4000	-
3	GM130	Abcam	1:4000	-
4	Cytochrome-C	Abcam	1:4000	-
6	IL4R	Santacruz	1:3000	1:200
7	Anti-rabbit secondary	Cell Signaling	1:10000	-
8	Anti-mouse secondary	Cell Signaling	1:10000	-
9	FITC conjugated Anti-rabbit secondary	Cell Signaling	-	1:400
10	Alexa Fluor-647-anti-mouse secondary	Cell Signaling	-	1:400

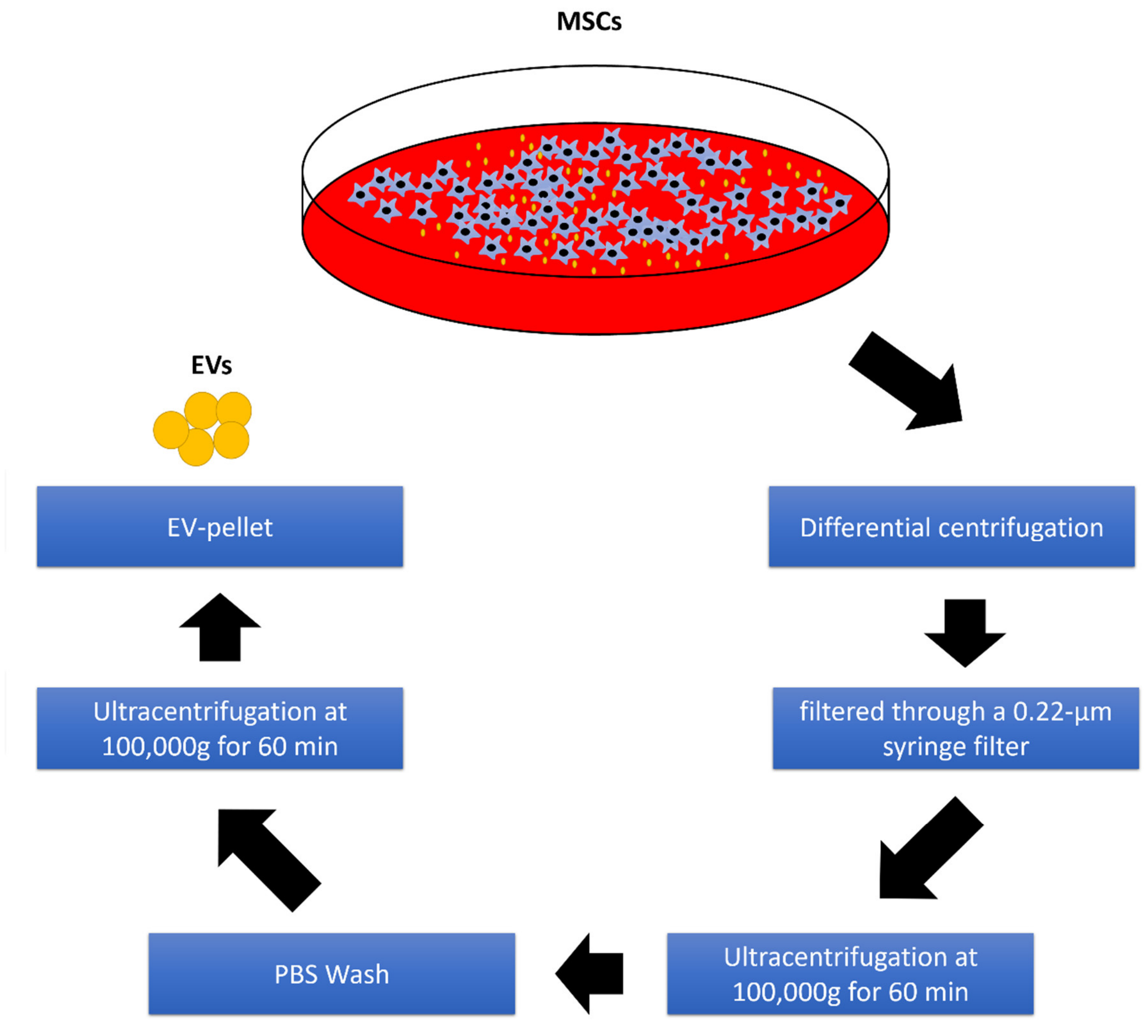

Supplementary Figure S1. Schematic representation of isolation of extracellular vesicles (EVs) from mesenchymal stem cells (MSCs).

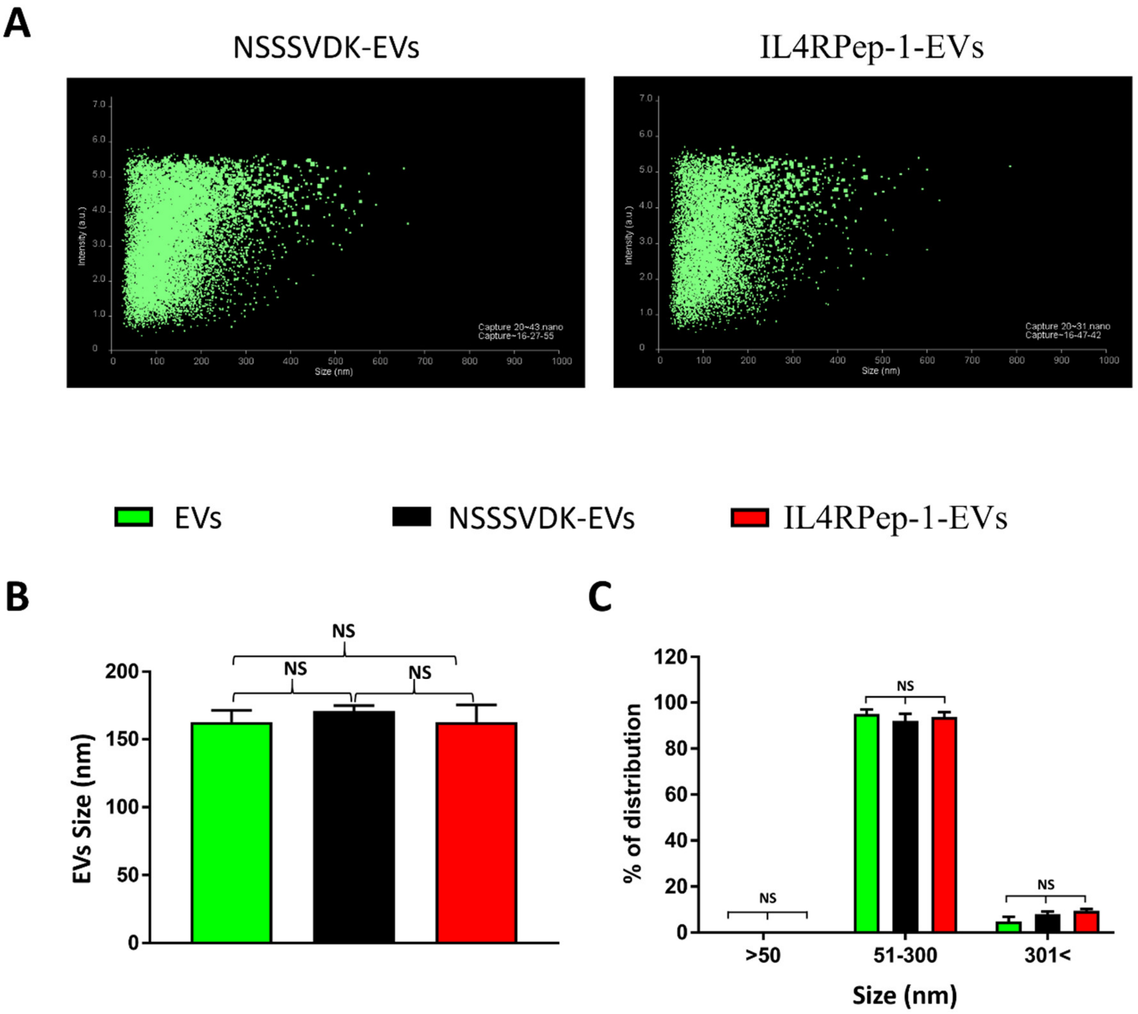

Supplementary Figure S2. Peptide labeling retains the size and distribution of extracellular vesicles (EVs). (A) Scatter plot of NSSSVDK-EV and IL4RPep-1-EV analyzed by nanoparticle tracking analysis (NTA). **(B)** Size of EVs, NSSSVDK-EV and IL4RPep-1-EV determined by NTA. **(C)** Percentage distribution of EVs represented on bar graph (Approximately 95 % EVs ranged in size between 50-300 nm). Data are represented as mean ± standard deviation (SD). Statistical difference between groups was analyzed using Student's t-test.

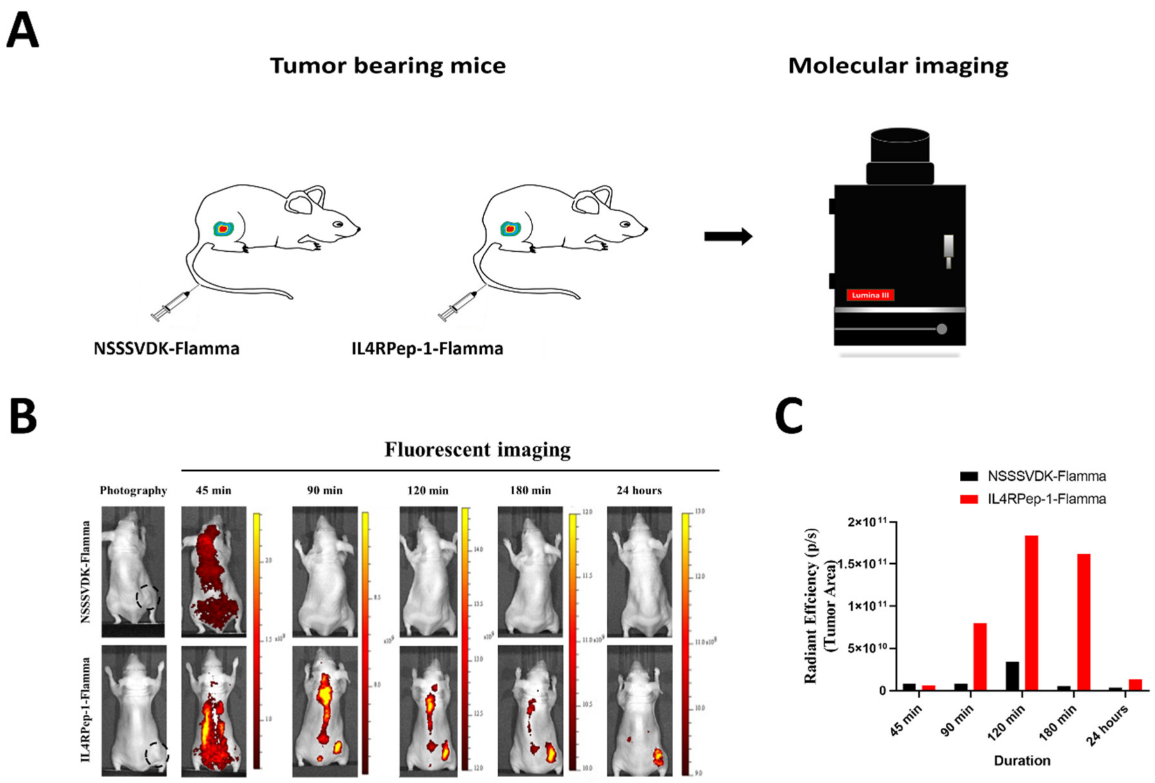

Supplementary Figure S3. Intravenously (i.v.) injected IL4R Pep-1 targets Cal-62 tumor in mouse (n=1/group), monitored by fluorescent imaging. (A) Schematic image of Cal-62 tumor bearing mouse i.v. injection with NSSSVDK-Flamma and IL4RPep-1-Flamma; mouse imaged with IVIS imaging system. **(B)** Florescent imaging at 45, 90, 120, 180 min and 24 h post-i.v. injection of NSSSVDK-Flamma or IL4RPep-1-Flamma in Cal-62 tumor bearing mouse. **(C)** Quantification of florescent signals at the tumor region.
